# HisCoM-PAGE: software for hierarchical structural component models for pathway analysis of gene expression data

**DOI:** 10.5808/GI.2019.17.4.e45

**Published:** 2019-12-23

**Authors:** Lydia Mok, Taesung Park

**Affiliations:** 1 Interdisciplinary Program in Bioinformatics, Seoul National University, Seoul 08826, Korea; 2 Department of Statistics, Seoul National University, Seoul 08826, Korea

**Keywords:** gene expression, hierarchical component model, pathway analysis, survival phenotype

## Abstract

To identify pathways associated with survival phenotypes using gene expression data, we recently proposed the hierarchical structural component model for pathway analysis of gene expression data (HisCoM-PAGE) method. The HisCoM-PAGE software can consider hierarchical structural relationships between genes and pathways and analyze multiple pathways simultaneously. It can be applied to various types of gene expression data, such as microarray data or RNA sequencing data. We expect that the HisCoM-PAGE software will make our method more easily accessible to researchers who want to perform pathway analysis for survival times.

**Availability:** HisCoM-PAGE is available on the website (http://statgen.snu.ac.kr/software/HisCom-PAGE/).

## Introduction

Transcriptome profiling is a common approach to the study of human diseases [1]. The transcriptome contains information about the RNA transcribed from the genome in a certain physiological or pathological condition [2,3]. Therefore, gene expression profiling can be applied to diagnose diseases, to predict their prognosis, and to select an appropriate therapy [3]. Many gene expression analyses have been conducted to find differentially expressed genes (DEGs) associated with a certain disease. However, gene expression analysis at the pathway level has the following advantages. First, mapping thousands to tens of thousands of genes into a few hundred pathways can reduce the problems associated with multiple testing [4]. Furthermore, researchers may obtain more interpretable results, compared to single-gene lists such as DEGs [5,6].

Many previous pathway analyses have mainly focused on binary phenotypes. Thus, not many methods and software programs are available to analyze the survival phenotype. In addition, many pathway methods perform single-pathway analyses, but do not consider correlations between pathways.

Recently, we proposed the hierarchical structural component model for the pathway analysis of gene expression data (HisCoM-PAGE) method [7] based on our previous work [8-10]. The HisCoM-PAGE method was proposed to identify significant pathways that are associated with the prognosis of complex diseases such as cancer, and it can consider correlations among pathways. In order for the HisCoM-PAGE method to be more accessible to researchers, we have made the HisCoM-PAGE software available on a dedicated website.

## Implementation

**Fig. 1.**
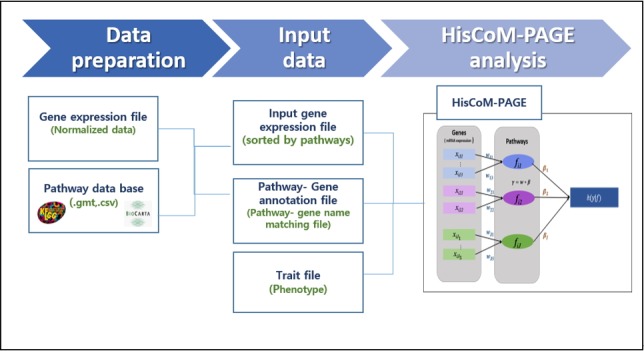
The workflow of hierarchical structural component model for pathway analysis of gene expression data (HisCoM-PAGE). The pathway is constructed by a weighted sum of its mapped genes. The pathway coefficient is represented as β and the gene coefficient is represented as w in the Figure.

The workflow of the HisCoM-PAGE software is shown in Fig. 1. The HisCoM-PAGE software requires an mRNA expression dataset and two other additional files (survival phenotype file.csv [or .rds], pathway annotation file.csv). The pathway annotation file can be obtained from a public pathway database. Genes should be mapped to pathways using pathway databases such as the Kyoto Encyclopedia of Genes and Genomes (KEGG) or the Biocarta database [11,12]. In this step, users can select the pathway database. After constructing a gene-pathway pair set, HisCoM-PAGE can be performed. HisCoM-PAGE implements a double-ridge method to analyze multiple pathways [13]. Cross-validation is used to find the optimal tuning parameters [14]. The HisCoM-PAGE software is entirely written in R code.

### Input files

The HisCoM-PAGE software takes the following three inputs: (1) a gene expression file, where the rows represent the sample names, the columns represent the pathway-matched gene name, and each cell represents the normalized gene expression value; (2) a trait file, in which each line consists of two columns for the sample’s survival time and censoring status, respectively; and (3) a pathway annotation file consisting of two columns for pathway and gene names, respectively. Users can optionally make their own gene-pathway pair list for analysis.

### Output file

The HisCoM-PAGE software can generate the following three output files: (1) a ‘pathway results.csv’ file, which contains four columns for the pathway name, pathway coefficient (β_path_), permutation p-value, and the false discovery rate (FDR) corrected q-value, respectively; (2) a ‘gene result-1.csv’ file containing five columns showing the pathway name, gene name, gene coefficient (*w*_gene_), p-value, and FDR-corrected q-value, respectively (the coefficients contained in this gene result-1 file relate to the weighting values that represent the effect of various genes on the pathway); and (3) a ‘gene result-2.csv’ file that also contains five columns showing the pathway name, gene name, gene coefficient, p-value, and FDR-corrected q-value, respectively. The coefficients contained in this result file are calculated using the *w*_gene_× β_path_ value for each gene, which represents the effect of the gene on the phenotype.

## Conclusion

In this paper, we introduce the HisCoM-PAGE software for pathway analysis of the survival phenotype using gene expression data. The HisCoM-PAGE software may be a useful tool for the identification of pathways associated with the survival phenotype. The software is freely available on the website, along with a detailed tutorial.
